# The Effect of Herbal Medicinal Products on Psoriasis-Like Keratinocytes

**DOI:** 10.3390/biom11030371

**Published:** 2021-03-02

**Authors:** Fabian Gendrisch, Birgit Haarhaus, Nina Krieger, Karl-Werner Quirin, Christoph M. Schempp, Ute Wölfle

**Affiliations:** 1 Research Center skinitial, Department of Dermatology, Medical Center, Faculty of Medicine, University of Freiburg, 79106 Freiburg, Germany; Fabian.Gendrisch@uniklinik-freiburg.de (F.G.); birgit.haarhaus@uniklinik-freiburg.de (B.H.); nina.krieger@uniklinik-freiburg.de (N.K.); Christoph.Schempp@uniklinik-freiburg.de (C.M.S.); 2 Flavex Naturextrakte GmbH, 66780 Rehlingen, Germany; wq@flavex.com

**Keywords:** psoriasis, interleukin 17A, interleukin 22, inflammation, β-defensin 2, *Humulus lupulus*, *Hypericum perforatum*, *Curcuma amada*

## Abstract

Psoriasis is a chronic inflammatory skin disease characterized by hyperproliferation of keratinocytes and expression of pro-inflammatory cytokines in the epidermis. New biological drugs were developed for the systemic treatment of moderate to severe psoriasis. However, products for the topical treatment of mild psoriasis are still required. Here, we examined the effect of natural compounds on psoriasis-like keratinocytes in vitro and ex vivo. Psoriasis-like keratinocytes were generated by treating human primary keratinocytes with the psoriasis-associated cytokines IL-17A, TNF-α and IL-22. Initially, 10 botanical extracts from Ayurvedic Medicine, Traditional Chinese Medicine, Northern American traditional medicine and Occidental Monastic Medicine were investigated using BrdU assays and IL-6 and IL-8 ELISAs. *Curcuma amada*, *Humulus lupulus* and *Hypericum perforatum* turned out to be the most effective plant extracts. In vitro, the plant extracts inhibited the expression of anti-microbial peptides (β-defensin 2), the hyperproliferation marker keratin 17, the glucose transporter 1 and downregulated the nuclear translocation of NF-κB and pSTAT3. In an ex vivo psoriasis model, *Humulus lupulus* displayed the most prominent anti-proliferative and anti-inflammatory effect. In conclusion, among the plant extracts investigated, *Humulus lupulus* showed the most promising anti-psoriatic effect. It is an interesting candidate for topical psoriasis treatment that should be further studied in clinical trials.

## 1. Introduction

Psoriasis is a chronic inflammatory skin disease that also frequently affects the joints and is associated with other comorbidities such as obesity and vascular diseases. In Central Europe, about 2–3% of the population suffers from psoriasis, with around 125 million cases worldwide: 65–80% of all psoriasis patients have a mild form of psoriasis and use topical products [1]. Topical therapies should clear small skin lesions, prevent the recurrence of psoriasis plaques and have no or only minimal side effects. The most effective topical compounds are corticosteroids and the anthracene derivative small molecule dithranol. However, the long-term use of topical corticosteroids may result in skin fragi-lity and dithranol is mainly used in clinical settings due to a difficult outpatient handling. Therefore, there is a high need for new safe and effective topical compounds. This study aimed to find a botanical extract that meets these requirements.

Several cell types such as dendritic cells (DC), T cells, keratinocytes, neutrophils, mast cells and macrophages are involved in the pathogenesis of psoriasis [2]. In our experiments, we focused on keratinocytes because they play an important role in both initiating and maintaining the psoriatic inflammation and simultaneously showing a psoriatic phenotype. In healthy persons, there exists a strictly regulated balance between proliferation of basal keratinocytes and desquamation of corneocytes, while psoriasis is characterized by an imbalance of this complex homeostasis [3]. In psoriasis, hyperproliferation of keratinocytes results in disturbed cell differentiation, eventually leading to thickening and scaling of the epidermis. Overexpression of epidermal keratin 17 (KRT17) is a typical marker of hyperproliferation in psoriatic skin [4]. Keratinocytes are also involved in the immunopathogenesis of psoriasis. Psoriatic lesions are frequently triggered by unspecific insults like trauma or chemical irritants leading to the release of self-DNA and RNA from dead or damaged keratinocytes. These nucleotides form complexes with anti-microbial peptides in the skin (e.g., LL-37) and activate innate immune cells like neutrophils and DC via Toll-like receptor (TLR) 9 [5,6]. Activated neutrophils release pro-inflammatory reactive oxygen species (ROS) and DC produce TNF-α and interleukin (IL)-23. IL-23 is considered to be a key cytokine in psoriasis that promotes the proliferation of skin-resident T helper cells (especially Th17 and Th22) and the release of IL-17A and IL-22. Psoriatic keratinocytes also produce chemokines (e.g., CCL20 and CXCL8 (IL-8)), anti-microbial peptides (β- defensin 2 (DEFB4A) and psoriasin (S100A7)) and other inflammatory factors (TNF-α, IL-6, IFN-γ). The secretion of these factors recruits additional Th1, Th17 and Th22 cells and neutrophils into the skin, and amplifies the inflammatory IL17/IL23 pathway [6,7]. This inflammatory crosstalk between keratinocytes and immune cells is responsible for the induction and maintenance of the psoriasis phenotype [7]. The psoriasis-associated cytokines IL-17A, IL-22 and IFN-γ induce the expression of the transcription factor Nrf2 and subsequently KRT17, eventually leading to hyperproliferation of keratinocytes [8].

In this study, we investigated 10 plant extracts that are characterized in Table 1. All these extracts are traditionally used in folk medicine in eastern and western Asia (Traditional Chinese Medicine, TCM; *Centella asiatica*), in the Indian subcontinent (Ayurveda medicine, AM; *Bacopa monniera, Centella asiatica, Commiphora mukul, Curcuma amada, Humulus lupulus* and *Whrightia tinctoria*), in America (Northern American traditional medicine; *Echinacea purpurea*) and in Europe (Occidental Monastic Medicine, OMM; *Gentiana lutea, Humulus lupulus*, *Hypericum perforatum*, *Menyanthes trifoliata*), with possible effectiveness against psoriasis from their traditional use. *Centella asiatica, Commiphora mukul*, *Gentiana lutea, Hypericum perforatum* and *Wrightia tinctoria* are already used for psoriasis, but dose-response studies and proof of concept studies are missing. Anti-proliferative effects of these extracts were often reported from experiments with tumor cells (e.g., in *Curcuma amada*) and it is unclear if these effects can be transferred to psoriasis. The different lead compounds of these plant extracts include terpenes and terpenoides, steroids, phloroglucinol derivatives and nitrogen-containing alkylamides, and are summarized in Table 1.

The various pharmacological effects of these compounds, including anti-inflammatory, anti-oxidative and anti-proliferative effects, are also listed in Table 1. Furthermore, some extracts inhibit the AKT pathway (*Curcuma amada*, *Hypericum perforatum*, *Commiphora mukul*) or NF-κB translocation (*Gentiana lutea*, *Commiphora mukul*, *Humulus lupulus*, *Echinacea purpurea*). NF-κB can bind to the promotor of several pro-inflammatory cytokines such as IL-1β, IL-6 and TNF-α [47] that are involved in the pathogenesis of psoriasis.

In a first screening approach, the 10 extracts were tested for the reduction of proliferation and pro-inflammatory signaling pathways that are characteristic for psoriatic keratinocytes. The most effective plant extracts, *Humulus lupulus* (HL), *Hypericum perforatum* (HP) and *Curcuma amada* (CA), were then tested for their effect on the JAK/STAT3 pathway and the marker of hyperproliferation in psoriasis, KRT17, that is regulated by the transcription factor NF-κB. In addition, the expression of the psoriasis marker *DEFB4A*, coding for the anti-microbial peptide β-defensin 2, was investigated.

## 2. Materials and Methods

### 2.1. Plant Extracts

All extracts were produced by Flavex Naturextrakte GmbH. The dried botanicals were gently milled to a powder with typical particle size between 200 and 600 microns by using a cutting mill in a few cases, followed by a pin or turbo mill as a second step. The guggul gum resin was deep-frozen, passed through a cutting mill and blended in a ratio of 1 + 1 with kieselguhr to form a powder.

High-Pressure Ethanol (HPE) extraction was archived by percolation of the plant powder in a pressure vessel with ethanol at 100 bar, 80 °C and 10 kg/kg solvent ratio. The ethanol was completely removed by vacuum distillation, and in case of *Menyanthes trifolia* and *Gentiana lutea*, the remaining extract was again mixed with ethanol in a ratio of 1 + 1. Whereas HPE extraction removes constituents of medium polarity like glycosides, supercritical CO_2_ extraction even with a small amount of ethanol as co-solvent (used for *Commiphora mukul*) is a non-polar solvent extracting lipophilic compounds only. The 3 favourites from efficacy screening, *Humulus lupulus* (HL), *Hypericum perforatum* (HP) and *Curcuma amada* (CA) were so-called total CO_2_ extracts, i.e., all components soluble in pure CO_2_ were received as extract. Accordingly, the extract represents the complete lipophilic spectrum of plant constituents without any solvents and dilution, obtained under gentle process conditions.

HL and CA were extracted at 280 bar/45 °C and HP at 300 bar/40 °C. The CO_2_ amount was adjusted to obtain an extraction degree of about 90% of marker constituents based on the mess balance of markers in feedstock, extract and extraction residue. The active components of all extracts were analysed by HPLC. Authentic reference substances were used for identification and quantification. In case of HL (which does not contain Xanthohumol), the International Calibration Extract (ICE) 3 (Veritas AG, Zürich, Switzerland) was used for analyzing alpha and beta acids; in case of HP (which does not contain hypericin), a self-isolated hyperforin/adhyperforin standard, concentration verified by Phytolab, Germany, was used, and in case of CA, a self-isolated (E)-Labda-8(17),12-diene-15,16-dial (LDD) standard was used according to Reference [48]. The characteristics of all extracts are summarized in Supplementary Table S1.

### 2.2. Antibodies and Reagents

The following antibodies and dilutions were used for immunohistochemical stainings: anti KRT17 (abcam, Berlin, Germany, 1:200), anti-BD 2 antibody (Peprotech, Rocky Hill, CT, USA, 1:100), anti-psoriasin antibody (abcam, Cambridge, MA, USA, 1:100), anti-GLUT1 antibody (abcam, Cambridge, MA, USA, 1:200), anti-NF-κB p65 antibody (F-6) (Santa Cruz, Heidelberg, Gemany) and the phospho-STAT3 (Tyr705; Cell Signaling Technologies, Leiden, the Netherlands). The secondary antibody multi-link-biotin (Agilent-Dako, Hamburg, Germany, 1:200), the streptavidin-HRP-label (abcam, Cambridge, MA, USA) and the AEC-substrate (Zytomed, Berlin, Germany) were used according to the manufacturer’s protocol. The fluorescence secondary antibodies Alexa Fluor 555 goat anti-mouse IgG and Alexa Fluor 555 donkey anti-rabbit IgG were from Thermo Fisher Scientific (Dreieich, Gemany). IL-22, IL-17A and TNF-α were from Peprotech (Rocky Hill, CT, USA). Dithranol and DAPI (4′,6-Diamidino-2-phenylindole dihydrochloride) were from Sigma-Aldrich GmbH (Taufkirchen, Germany).

### 2.3. Cell Culture

Human primary keratinocytes (HPK) were prepared from adult skin of reduction surgery (approved by the ethics committee of the University Medical Center Freiburg, Certificate No EK432/18) and cultured according to the method of Rheinwald and Green [49] in Keratinocyte-SFM medium (Thermo Fisher, Darmstadt, Germany). To generate psoriasis-like HPK, cells were first grown in basal Keratinocyte-SFM medium (Thermo Fisher, Darmstadt, Germany) and then incubated with psoriasis cytokines (IL-17A, IL-22 and TNF-α, 20 ng/mL each) for 24 h. All cells were cultured at 37 °C in a humidified atmosphere with 5% CO_2_. For extract treatment, the cells were incubated with the extracts (1 µg/mL HL (*Humulus lupulus*), 1 µg/mL HP (*Hypericum perforatum*), 2 µg/mL CA (*Curcuma amara*)) or 0.3 µg/mL dithranol for 2 h before the additional stimulation with the psoriasis cytokines was performed for 24 h.

### 2.4. Cell Viability Assay

HPK were seeded in a 96-well plate (6000 cells per well) over night and were incubated with basal Keratinocyte-SFM medium (Thermo Fisher, Darmstadt, Germany) for 24 h. Then, the cells were incubated for a further 24 h with the plant extracts before the cell viability was assessed with the CellTiter-Glo2.0 Assay (Promega, Walldorf, Germany) according to the manufacturer’s protocol. The method is based on the bioluminescent measurement of ATP that is present in metabolically active cells. Luciferase catalyzes the formation of light from ATP and luciferin. The emitted light intensity is linearly related to the ATP concentration and is measured using a scanning multiwell spectrophotometer (Sirius HT from BioTek, Bad Friedrichshall, Germany).

### 2.5. Cell Proliferation (BrdU Assay, Roche)

Proliferation was assessed by the BrdU cell proliferation assay (Roche, Mannheim, Germany). 2 × 10^3^ HPK were seeded per well in a 96-well plate and incubated for 24 h at 37 °C under 5% CO_2_. Then, the cells were incubated with basal Keratinocyte-SFM medium (Thermo Fisher, Darmstadt, Germany) for 24 h and stimulated for 72 h with psoriasis cytokines (IL-17A, IL-22 and TNF-α, 20 ng/mL each) before the plant extracts were added for 24 h. The BrdU assay labeling occurred according to the manufacturer’s instruction. Absorption at a wavelength of 450 nm was measured in a scanning multiwell spectrophotometer (Sirius HT from BioTek, Bad Friedrichshall, Germany).

### 2.6. RNA Extraction and RT-PCR

The mRNA expression levels of *DEFB4*, *KRT1*, *KRT17*, *GLUT1* and *ACTB* were measured by real-time qRT-PCR. Total RNA was extracted from HPK using the TRIzol reagent (Thermo Fisher, Darmstadt, Germany). After extraction, 1 µg of total RNA was reverse transcribed using the iScript cDNA Synthesis Kit (BIO-RAD, Feldkirchen, Germany) to obtain cDNA. The real-time qRT-PCR reaction was performed using a SYBR Green kit (Thermo Maxima SYBR Green qPCR Master Mix, Thermo Fisher, Darmstadt, Germany) on a Bio-RAD CFX96^TM^ Real-Time System. The used primers were the following:

Hu DEFB4 5′-ACCACCAAAAACACCTGGAAG-3′ forward and 5′-ACCAGGGACCAGGACCTTTA-3′ reverse; hu KRT1 5′-GGCAGACATGGGGATAGTGTG-3′ forward and 5′-CTTGAGGGCATTCTCGCCA-3′ reverse; hu KRT17 5′-GAGATTGCCACCTACCGCC-3′ forward and 5′-ACCTCTTCCACAATGGTACGC-3′ reverse; hu GLUT1 5′-TCTGGCATCAACGCTGTCTT-3′ forward and 5′-AAGGCAAGTGTCTCGACAGG-3′ reverse; hu ACTB: 5′-CACTGTCGAGT CGCGTCC-3′ forward and 5′-TCATCCATGGCGAACTGGTG-3′ reverse. The relative gene expression was determined using the comparative C_T_ method using the formula of 2^−ΔΔCt^ [50] with *ACTB* as the internal control. Data were expressed as mean ± standard deviation (SD) of three independent experiments from 3 different skin biopsies.

### 2.7. Immunohistochemistry

Three µm sections of paraffin-embedded skin explants were stained for keratin 17 (KRT17), S100A7 (Psoriasin), glucose transporter 1 (GLUT1) and β-defensin 2 (BD2) with the above-listed primary and secondary antibodies using the LSAB method (DCS, Hamburg, Germany). Random fields of sections from ≥2 independent skin explants were photographed at 100x magnification.

### 2.8. Immunofluorescence

HPK were pre-treated with either 1 µg/mL HL (*Humulus lupulus*), 1 µg/mL HP (*Hypericum perforatum*), 2 µg/mL CA (*Curcuma amada*) or 0.3 µg/mL dithranol for 2 h before the additional stimulation with the psoriasis cytokines (20 ng/mL of IL-17A, IL-22 and TNF-α) was performed for 24 h. Then, the cells were fixed using 4% formaldehyde at RT for 10 min and permeabilized with methanol at −20 °C for 10 min. Subsequently, the cells were blocked with 5% BSA for 1 h at RT and incubated overnight at 4 °C with the primary antibodies (anti-p65 subunit of NF-κB antibody and anti-pSTAT3 antibody). DAPI was used for nuclear staining. The % of total protein located in the nucleus was measured using the Intensity Ratio Nuclei Cytoplasm Tool (RRID:SCR_018573) in ImageJ. Data were shown as mean ± SD of five independent experiments with two pictures per experiment.

### 2.9. IL-6 and IL-8 ELISA

IL-6 and IL-8 concentrations in cell culture supernatants were analyzed by a high-sensitivity IL-6 and IL-8 ELISA (BD, San Jose, CA, USA) according to the manufacturer’s protocol. Data were expressed as mean ± SD of three independent experiments from 3 independent HPK or skin samples.

### 2.10. Psoriasis Ex Vivo Model

Ex vivo skin explants were prepared from sterile human skin biopsies (approved by the ethics committee of the University Medical Center Freiburg (Certificate No EK432/18). In brief, subcutaneous fat was removed to receive skin samples containing epidermis and papillary dermis. The skin was blot-dried and tape-stripped (5 times) with autoclavable stripes. Then, the skin was punched in 8 mm skin pieces and placed on cell culture inserts with 1.0 µm pore size (BD Falcon, Heidelberg, Germany), which were placed in 12-well plates (BD Falcon, Heidelberg, Germany). Skin punches were cultured at the air–liquid interphase and cultured under standard cell culture conditions. The culture medium contained DMEM/Keratinocyte-SFM medium at equal concentrations with 5% FCS, 1.2 mM CaCl_2_ and 50 µg/mL ascorbic acid. Round filter paper (8 mm) was soaked with 40 µL plant extract (1 µg/mL HL (*Humulus lupulus*), 1 µg/mL HP (*Hypericum perforatum*), 2 µg/mL CA (*Curcuma amada*) or 0.4 µg/mL dithranol) and placed on top of the skin explants for 24 h. Subsequently, the explants were stimulated with psoriasis cytokines (IL-17A, TNF-α, IL-22, 20 ng/mL each) in the medium. The medium was changed daily, and psoriasis cytokines and ascorbic acid were freshly added to the medium and the filter paper was also exchanged daily. After 4 days, the explants were harvested. The supernatants were snap-frozen in liquid nitrogen and stored at −80 °C until being used for IL-6 and IL-8-ELISA. The skin explants were fixed in formaldehyde, embedded in paraffin and stored at room temperature for immunohistochemical stainings.

### 2.11. Statistical Analysis

Data analysis was performed using GraphPad Prism version 6.0 software (GraphPad Software, San Diego, CA, USA). Significant statistical differences were evaluated using one-way analysis of variance (ANOVA) followed by the Newman-Keuls Test or the column statistic with one sample t-test in case the stimulated sample was set as 100%*. p*-values of <0.05 were considered statistically significant. Data are presented as the means ± standard deviation (SD) of at least three independent experiments from three skin samples.

## 3. Results

### 3.1. In Vitro Psoriasis Model

Several cytokines are used for the generation of psoriasis-like HPK, i.e., IL-17A, TNF-α, IL-36γ, oncostatin, IFN-γ, IL-22 and IL-1α [51,52]. We treated HPK with different combinations of cytokines for different time periods. We then analyzed the cells for markers typical for psoriasis, e.g., the expression of DEFB4A (coding for β-defensin 2), as well as the increase of the pro-inflammatory cytokines IL-6 and IL-8. We obtained a psoriasis-like phenotype of HPK with a combination of physiological concentrations of IL-17A, IL-22 and TNF-α (20 ng/mL each). Especially IL-17A and TNF-α display an important inflammatory effect in the pathogenesis of psoriasis [53,54,55], whereas only IL-22, but not IL-17A and TNF-α, cause epidermal alterations typical for psoriasis such as acanthosis [54]. The psoriasis cytokine mix induced a psoriasis phenotype already after 24 h.

The increased expression of DEFB4A in the skin of psoriasis patients has been described earlier [56] and was recently confirmed to be a good biomarker of psoriasis [57]. Therefore, we analyzed its expression in HPK after treatment with the above-described cytokines.

**Figure 1:**
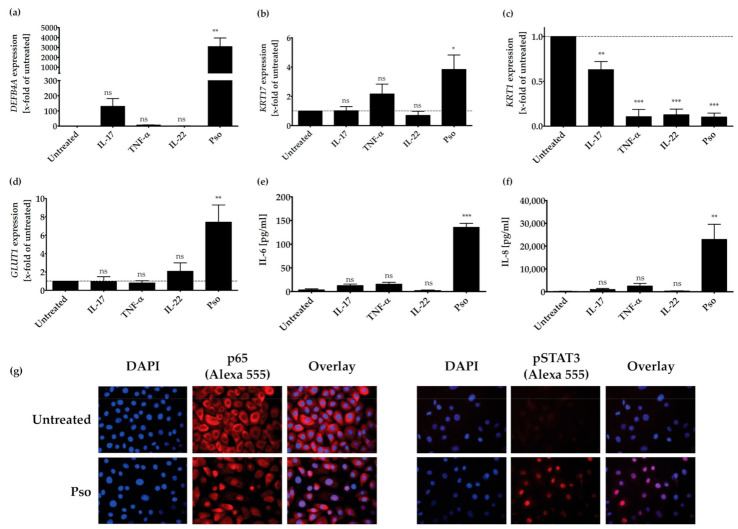
IL-17A-, TNF-α- and IL-22-induced synergy by expression of psoriasis-related genes or proteins. HPK were stimulated with IL-17A (20 ng/mL), TNF-α (20 ng/mL), IL-22 (20 ng/mL) or a combination of these cytokines (Pso) for 24 h. (**a**) *DEFB4A*, (**b**) *KRT17*, (**c**) *KRT1* and (**d**) *GLUT1* mRNA expression was analyzed by real time qRT-PCR (*n* = 3). *ACTB* was used as reference gene for normalization. Protein level of secreted IL-6 (**e**) and IL-8 (**f**) was measured in the cell culture supernatant by ELISA (*n* = 3). The dotted line indicates the value of the untreated sample. Data shown as mean ± SD of three independent experiments. (**g**) HPK were stimulated with the cytokine combination (20 ng/mL of IL-17A, IL-22 and TNF-α, Pso) for 24 h and immunofluorescence stainings of p65 NFκB and pSTAT3 were performed. The nucleus is stained with DAPI. Representative pictures of p65 NFκB and pSTAT3 staining in untreated and cytokine-treated cells. The % of total protein in the nucleus was measured using the Intensity Ratio Nuclei Cytoplasm Tool (RRID:SCR_018573) in ImageJ. Data shown as mean ± SD of five independent experiments with two pictures per experiment. Statistics were always one-way ANOVA with Newman-Keuls post-test. * *p* ≤ 0.05, ** *p* ≤ 0.01, *** *p* ≤ 0.001, ns-not significant (*p* > 0.1).

As seen in Figure 1, only the treatment of HPK with IL-17A resulted in an increase of *DEFB4A* expression. The combination of all three cytokines prominently increased *DEFB4A* expression (Figure 1a). As KRT17 plays a key role in promoting keratinocyte hyperproliferation, we assessed if the psoriasis cytokines were also able to increase KRT17 expression in HPK. As shown in Figure 1b, only the combination of IL-17A, TNF-α and IL-22 significantly increased KRT17 expression in relation to the untreated control (Figure 1b). Keratin 1 (KRT1) is directly involved in cell cycle control, which regulates keratinocyte differentiation [58]. It represents an early differentiation marker that is downregulated in psoriasis; therefore, we also investigated if the psoriasis cytokines can reduce *KRT1* expression. TNF-α or IL-22 alone as well as the cytokine combination significantly reduced *KRT1* expression, whereas IL-17A showed a weaker effect (Figure 1c). Proliferating cells are dependent on glucose for their growth. The glucose transporter-1 (GLUT1) is a member of sugar transporters and is necessary for injury- and inflammation-associated keratinocyte proliferation. This transporter is weakly expressed in healthy skin, but strongly upregulated in psoriasis lesional skin. Because it might facilitate epidermal hyperproliferation [59,60,61], we investigated if our cytokine mix has an effect on *GLUT1* expression. Again, only the combination of all three cytokines significantly increased the expression of *GLUT1* (Figure 1d).

Treatment of HPK with the single cytokines IL-17A, IL-22 or TNF-α had no significant effect on the production of the pro-inflammatory cytokines IL-6 and IL-8 (Figure 1e,f). However, a combination of all three cytokines strongly increased the production of IL-6 and IL-8. IL-6 [62] and IL-8 [63] play a central role in the pathogenesis of psoriasis, because they activate lymphocytes in psoriatic inflammation. In addition, the combination of psoriasis cytokines induced the nuclear translocation of the p65 subunit of NFκB and the phosphorylation of STAT3, as shown in immunocytochemical stainings (Figure 1g). Furthermore, the cytokine mix significantly induced the proliferation of HPK, as measured with the BrdU assay (Supplementary Figure S1a) without notable cytotoxic effects (the cytotoxicity lies below 5%, Supplementary Figure S1b). In summary, treatment of HPK with the cytokines IL-17A, IL-22 and TNF-α results in a psoriasis-like phenotype.

### 3.2. Screening of Plant Extracts for Anti-Psoriatic Effects In Vitro

Psoriasis-like HPK were screened for the anti-proliferative and anti-inflammatory potential with the 10 outlined plant extracts in Table 1 using BrdU assays and IL-6 and IL-8 ELISA. Only extracts of *Menyanthes trifoliata* L (buckbean), *Commiphora mukul* (guggul), *Humulus lupulus* (hop), *Hypericum perforatum* (St John’s wort) and *Curcuma amada* (mango ginger) displayed dose-dependent anti-proliferative and anti-inflammatory effects with half maximal effective concentrations (EC_50_) below 50 µg/mL (Supplementary Table S1). A cut-off of 50 µg/mL extract was defined due to practical considerations as higher concentrations may lead to drawbacks in topical applications. *Humulus lupulus* (HL), *Hypericum perforatum* (HP) and *Curcuma amada* (CA) turned out to be the most promising candidates, because these extracts were 10-times more effective than *Menyanthes trifoliata* L (buckbean) and *Commiphora mukul* (guggul).

The EC_50_ values for anti-proliferative and anti-inflammatory effects in psoriasis-like HPK range between 0.36 ± 0.002 and 2.33 ± 0.63 µg/mL (Table 2). The gold standard of topical psoriasis therapy, dithranol, has an EC_50_ value of 0.03 ± 0.001 µg/mL. The determined concentrations were not cytotoxic for HPK, as demonstrated by the EC_80_ value in the cell viability assay showing 80% viable cells.

### 3.3. Pathways Involved in the Anti-Psoriatic Effect of Curcuma amada (CA), Humulus lupulus (HL) and Hypericum perforatum (HP)

The effect of HL, HP and CA on gene expression of the hyperproliferation marker KRT17, the anti-microbial peptide DEFB4A, the early differentiation marker KRT1 and the glucose transporter GLUT1 were analyzed in psoriasis-like HPK. As HPK isolated from different skin biopsies varied in their stimulation capacity, the effect of extract treatment was shown in relation to the stimulated sample that was set as 100%. In contrast, reactions from the same biopsy were very matchable. Gene induction ranged for DEFB4A from 730- to 7684-fold, for KRT17 from 3.6- to 6.4-fold, for KRT1 from 0.12- to 0.27-fold and for GLUT1 from 2.2- to 7.12-fold.

**Figure 2:**
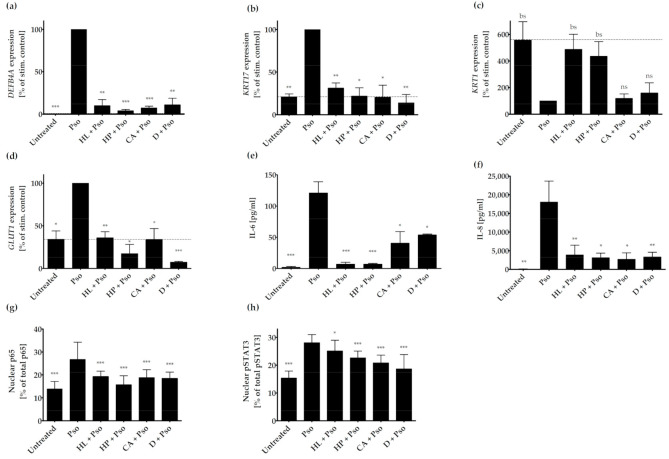
Effect of plant extracts on psoriasis-related genes or proteins. HPK were incubated with or without plant extracts (1 µg/mL HL (*Humulus lupulus*), 1 µg/mL HP (*Hypericum perforatum*), 2 µg/mL CA (*Curcuma amara*) or 0.3 µg/mL D (dithranol) for 2 h and then stimulated with psoriasis cytokines (IL-17A, TNF-α and IL-22; 20 ng/mL each) for 22 h (Pso). (**a**) DEFB4A, (**b**) KRT17, (**c**) KRT1 and (**d**) GLUT1 mRNA expression was analyzed by real time qRT-PCR (*n* = 3). ACTB was used as reference gene for normalization. Protein level of secreted IL-6 (**e**) and IL-8 (**f**) was measured in the cell culture supernatant by ELISA (*n* = 3). The dotted line indicates the value of the untreated sample. Data shown as mean ± SD of three independent experiments. (**g**) Effect of plant extracts on p65 subunit of NF-κB and (**h**) pSTAT3 nuclear translocation. HPK were pre-treated with either 1 µg/mL HL (*Humulus lupulus*), 1 µg/mL HP (*Hypericum perforatum*), 2 µg/mL CA (*Curcuma amara*) or 0.3 µg/mL D (dithranol) for 2 h prior to stimulation with 20 ng/mL of IL-17A, IL-22 and TNF-α. Cells were fixed, permeabilized and the p65 subunit of NF-κB and pSTAT3 were stained using the antibodies noted in the Materials and Methods Section, while DAPI was used for nuclear staining. The % of total protein in the nucleus was measured using the Intensity Ratio Nuclei Cytoplasm Tool (RRID:SCR_018573) in ImageJ. Data shown as mean ± SD of five independent experiments with two pictures per experiment. Statistics were always one-way ANOVA with Newman-Keuls post-test. * *p* ≤ 0.05, ** *p* ≤ 0.01, *** *p* ≤ 0.001, bs (borderline significant): 0.1 > *p* > 0.05, ns-not significant (*p* > 0.1).

All three extracts significantly decreased gene expression of DEFB4A and KRT17 in a similar range as the positive control dithranol (Figure 2a,b). The early differentiation marker KRT1 was upregulated by HL or HP extract with borderline significant (bs) *p*-values of 0.07 and 0.09 for HL and HP respectively, whereas CA extract showed no effect (*p*-value: 0.66; Figure 2c). Dithranol also failed to induce KRT1 expression (*p*-value: 0.52; Figure 2c). However, all three extracts as well as dithranol reduced the expression of the glucose transporter GLUT1 (Figure 2d). Furthermore, all extracts and dithranol significantly reduced the expression and release of the pro-inflammatory cytokines IL-6 and IL-8 (Table 2 and Figure 2e,f).

Additionally, we analyzed the effect of the plant extracts on inflammation and proliferation-related pathways in psoriasis, such as STAT3 and NF-κB. The nuclear translocation of the NF-κB subunit p65 was reduced by all three plant extracts and dithranol (Figure 2g). pSTAT3 translocation was also inhibited by all compounds, as determined by the percentage of nuclear content of pSTAT3 (Figure 2h). However, the translocation of the phosphorylated protein kinase AKT was not reduced in extract-treated psoriasis-like HPK (data not shown).

### 3.4. Effects of Humulus lupulus, Hypericum perforatum and Curcuma amada in an Ex Vivo Psoriasis Model

In order to confirm the effect of HL, HP and CA in a more natural setting, a psoriasis ex vivo skin model was established. Skin punches from reduction surgeries harbor all cell types of the skin and were cultured for 4 days at the air–liquid interface in an appropriate differentiation medium [64]. By addition of the psoriasis cytokines from the in vitro experiments (IL-17A, TNF-α, IL-22, 20 ng/mL each), an inflammatory milieu typical for psoriasis was simulated.

As psoriatic lesions are frequently triggered by unspecific insults like a trauma or scratches, we performed tape stripping of the skin prior to taking punch biopsies. Subsequently, the secretion of the pro-inflammatory cytokines IL-6 and IL-8 was measured in the culture supernatant by ELISA.

**Figure 3:**
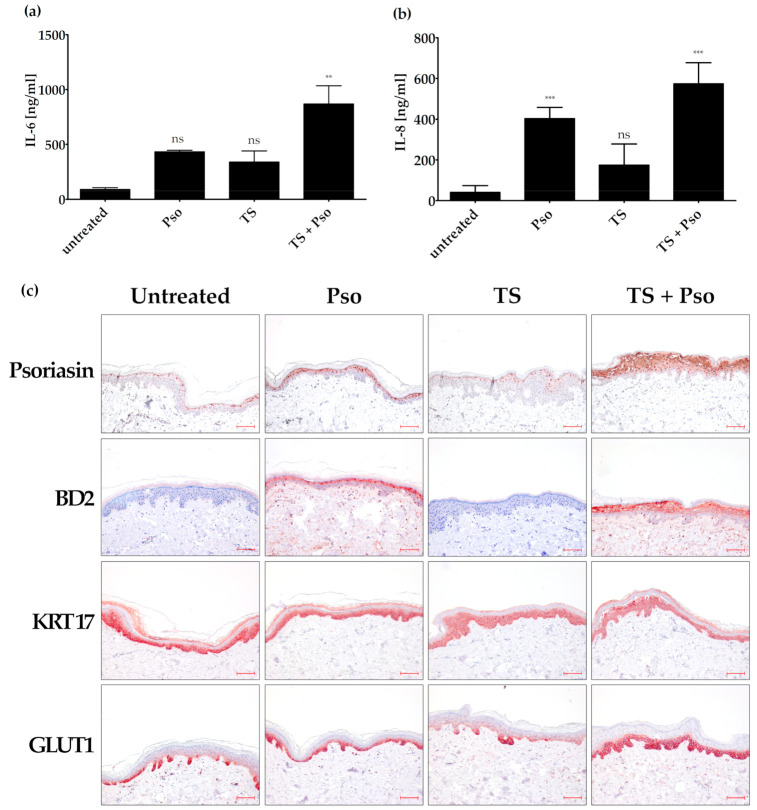
Generation of an ex vivo psoriasis model. Skin models have been cultured for 4 days at the air-liquid interface using appropriate differentiation medium. The skin models were left untreated, stimulated with psoriasis cytokines (IL-17A, TNF-α, IL-22, 20 ng/mL each; Pso), tape stripped (TS) or both actions were performed (TS + Pso). The protein level of secreted IL-6 (**a**) and IL-8 (**b**) was measured in the cell culture supernatant by ELISA (*n* = 4). (**c**) Skin models were fixed, embedded in paraffin and 3 µm sections were stained with antibodies against psoriasin (S100A7), β-defensin 2 (BD2), keratin 17 (KRT17) or glucose transporter 1 (GLUT 1) (*n* = 4). Scale bar = 100 µm. ** *p* ≤ 0.01, *** *p* ≤ 0.001, ns-not significant (*p* > 0.1).

Tape stripping together with the stimulation with psoriasis cytokines increased IL-6 expression compared to biopsies treated only with cytokines or tape stripping (Figure 3a). Psoriasis cytokines alone strongly increased the IL-8 expression in the skin samples. This effect could only be slightly increased by additional tape stripping. Tape stripping alone had a much smaller effect on IL-8 expression, although it was statistically significant (Figure 3b).

Immunohistochemical analysis of psoriasin (S100A7) also confirmed that tape stripping improves the ex vivo psoriasis skin model. Psoriasin is a psoriasis marker that controls cell proliferation and differentiation, has anti-microbial peptide activity and promotes the inflammatory process in psoriasis. The cultivation of skin biopsies for 4 days (untreated control) increased psoriasin expression compared to skin biopsies taken directly after surgery. In cytokine-stimulated skin, psoriasin expression in the nucleus and cytoplasm was increased in the upper layers of the skin (e.g., stratum granulosum and stratum corneum). Tape stripping had nearly no effect on psoriasin expression, whereas tape-stripped and cytokine-stimulated skin in all suprabasal layers showed a positive nuclear and cytoplasmic psoriasin staining (Figure 3c). This psoriasin staining is comparable to psoriatic skin with its weak expression in the basal cells and the intense expression in the nuclei and plasma membrane of the suprabasal skin layer [65].

β-defensin 2 (BD2), a further antimicrobial peptide, was strongly increased in our psoriasis ex vivo model. This protein is also expressed in lesional psoriasis skin, but not in healthy human skin. The untreated skin sample showed no BD2 expression. Treatment of skin punches with psoriasis cytokines increased the suprabasal BD2 expression, whereas tape stripping alone had no effect. Tape stripping together with psoriasis cytokines slightly increased BD2 expression compared to only cytokine-stimulated skin (Figure 3c).

As KRT17 upregulation alters cell proliferation, migration and inflammatory features that contribute to the hyperproliferation in psoriasis [66], we also analyzed KRT17 expression in our ex vivo psoriasis skin model. Unfortunately, KRT17 was already strongly expressed in the untreated skin sample in the whole epidermis (Figure 3c), and could not further be increased by cytokine stimulation and tape stripping. KRT17 together with KRT6 and 16 acts as an early barrier alarmin and is upregulated upon skin injury [66]. It is possible that this upregulation in our ex vivo psoriasis skin model is induced by the punch biopsy and handling procedures. Therefore, KRT17 is not a suitable marker in the ex vivo psoriasis skin model, although it was a good marker in the in vitro psoriasis model.

As GLUT1 showed an increased expression in our in vitro psoriasis model after stimulation with psoriasis cytokines, we also tested its role in our ex vivo psoriasis model. Cultivation of the skin model for 4 days showed a slight GLUT1 expression (Figure 3c). However, tape stripping together with cytokine stimulation further increased GLUT1 expression in the basal and first suprabasal cell layers (Figure 3c).

Taken together, the ex vivo psoriasis skin model generated by tape stripping and stimulation with psoriasis cytokines (IL-17A, TNF-α and IL-22) turned out as a useful psoriasis ex vivo model, with psoriasin, BD2 and GLUT1 as psoriasis markers.

**Figure 4:**
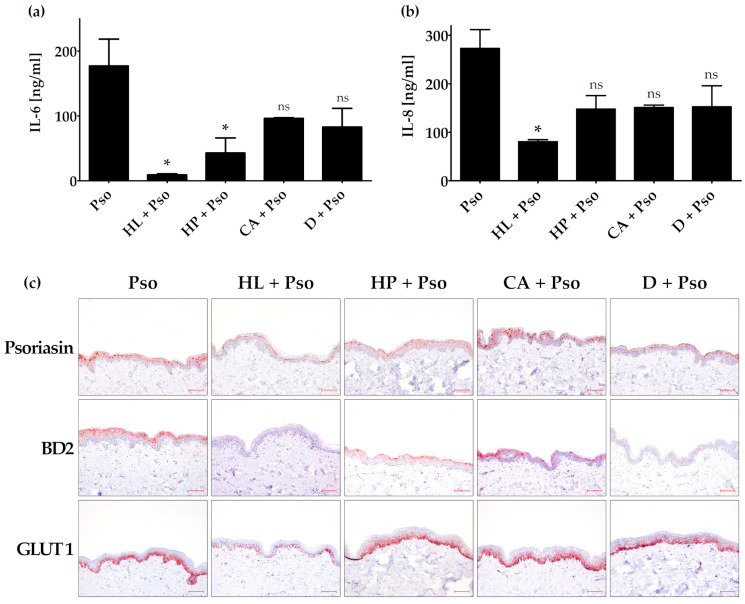
Effect of plant extracts in the ex vivo psoriasis model. The skin models were incubated with or without filter paper soaked with 40 µL plant extract solution on top of the skin explants (1.5 µg/mL HL (*Humulus lupulus*), 1.5 µg/mL HP (*Hypericum perforatum*), 3 µg/mL CA (*Curcuma amara*) or 0.4 µg/mL D (dithranol) for 24 h and then stimulated with psoriasis cytokines (IL-17A, TNF-α and IL-22; 20 ng/mL each (Pso)) for 72 h. The protein level of secreted IL-6 (**a**) and IL-8 (**b**) was measured in the cell culture supernatant by ELISA (*n* = 2). (**c**) Skin models were fixed, embedded in paraffin and 3 µm sections were stained with antibodies against psoriasin (S100A7), β-defensin 2 (BD2), keratin 17 (KRT17) or glucose transporter 1 (GLUT1). Scale bar = 100 µm. * *p* ≤ 0.05, ns-not significant (*p* > 0.1).

The ex vivo psoriasis skin model was treated with extracts of HL, HP and CA, or the positive control dithranol. When the extracts were added to the medium, no effect concerning the secreted IL-6 and IL-8 protein levels as well as the BD2, psoriasin and GLUT1 expression could be observed (data not shown). To simulate topical treatment, we applied the plant extracts or controls on top of the skin model via an extract-soaked filter paper. In this setting, we could demonstrate a significantly reduced expression and secretion of IL-6 after HL and HP treatment, whereas the treatment with CA and dithranol showed only a trend (Figure 4a).

Only HL treatment showed a significantly reduced IL-8 secretion. The other treatments were not statistically significant (Figure 4b). Treatment with HL and HP as well as dithranol could decrease psoriasin expression in the psoriasis ex vivo skin model, whereas treatment with CA showed no effect (Figure 4c). HL and dithranol strongly reduced BD2 expression in this psoriasis model. HP und CA also inhibited BD2 expression, but to a lesser extent. GLUT1 expression was only decreased by HL, whereas HP, CA and dithranol showed no effect (Figure 4c).

## 4. Discussion

During the last decade, several new biological drugs were developed for the systemic treatment of moderate to severe psoriasis and psoriatic arthritis. These immunological therapies neutralize the effect of various pro-inflammatory cytokines, such as TNF-α, IL-17A and IL-23. However, for the treatment of mild forms of psoriasis and childhood psoriasis, topical products are required. To date, prescription topical drugs include glucocorticoids, vitamin D derivatives, combinations of both and dithranol. All of these drugs may reduce symptoms such as pruritus and scaling and could prolong the symptom-free period, but also have limitations due to side effects such as skin fragility or skin irritation [67]. Herbal medicines are often less effective but have the advantage of fewer side effects. In addition, plant extracts possess more active compounds and therefore often act on various targets simultaneously. In this study, we aimed at identifying a plant extract that displays anti-psoriatic effects without severe toxic effects that could be further developed as a topical treatment for limited or childhood psoriasis. Several plant extracts have been described as anti-psoriatic in various in vitro test systems (*Artemisia capillaris* extract, paeoniflorin, curcumin, indigo, rhododendrin and isoflavones) [8,67,68,69,70,71,72,73]. However, most of the tests were performed with HaCaT cells with or without imiquimod (IMQ) stimulation. HaCaT cells represent an immortalized keratinocyte cell line and are considered as not very suitable for psoriasis research, because IL-17A or IFN-γ and TNF-α stimulation did not increase the expression of inflammatory markers (e.g., STAT3) or enhance cell proliferation in these cells [74]. On the other hand, psoriatic keratinocytes show increased cell proliferation, disturbed cell differentiation and an inflammatory phenotype, but they lose their specific psoriasis characteristics when they are no longer stimulated with psoriasis cytokines. Furthermore, the generation of psoriasis HPK cultures from psoriasis skin is difficult and only successful in about 10% of all cases [75]. Therefore, we established psoriasis-like HPK by treating HPK with the cytokines IL-17A, TNF-α and IL-22 [51,52]. Psoriasis-like HPK are very similar to cytokine-stimulated HPK isolated from psoriasis lesions.

The screening of ten plant extracts used in folk medicine showed that CA, HL and HP may reduce the proliferation of psoriasis-like HPK already at low concentrations. Exploring their mode of action revealed that these extracts downregulate KRT17 in vitro. KRT17 is a type I intermediate filament that is upregulated in psoriatic plaques and its expression indicates hyperproliferation under pathological conditions [66]. Treatment of human keratinocytes with IL17A, TNF-α and IL-22 upregulates KRT17 by the transcription factor STAT3 that is constitutively phosphorylated in psoriasis [76,77]. De Jong et al. compared the KRT17 expression in 6 psoriasis patients before and after topical treatment with dithranol or a vitamin D3 analogue [78]. They could show that KRT17 expression was reduced and positively correlated to the clinical response. Subsequently, it turned out that psoriasis treatments such as corticosteroids, retinoids and dithranol downregulate KRT17 [79,80,81,82]. In a mouse model, knocking down KRT17 inhibits the proliferation of keratinocytes in vitro and reduces epidermal hyperplasia and inflammatory cell infiltration on transplanted psoriatic tissue [66,83]. Therefore, it was suggested that KRT17 may be a critical factor in psoriasis pathogenesis and a good target for therapy [82]. The most promising extracts in our study (CA, HL and HP) also reduced the KRT17 expression in the in vitro psoriasis model. The results are comparable to studies with gallic acid, a natural small molecule from radix of *Paeoniae rubra*, that also suppressed IL17A-mediated KRT16 and 17 expression in HaCaT cells and in an IMQ-psoriasis mouse model in vivo [84]. This study showed comparable results in HaCaT cells with gallic acid in the same concentration range as the data described here. As the authors stimulated the cells only with IL-17A, the stimulatory range was quite small (1.3–2-fold induction concerning protein and mRNA expression). As our findings have shown, this induction might have been increased by the addition of TNF-α and IL-22.

Additionally, HL, HP and CA inhibited the activation of the transcription factor NF-κB, which can further influence the expression of KRT17 and is traditionally considered as a positive regulator of inflammation responsible for sustaining the inflammatory environment of psoriasis [72]. The expression of the psoriasis marker BD2 was also reduced by HL, HP and CA. BD2 acts not only as an anti-microbial peptide but it may also be involved in skin immunity by stimulating the expression of IL-6 or phosphorylation of the transcription factor STAT3 and promoting keratinocyte proliferation [85]. HL and HP, but not CA and the positive control dithranol, increased the expression of the differentiation marker KRT1 that is reduced in psoriasis-like HPK. Similarly, hyperforin, the major lipophilic active ingredient of HP, stimulates calcium influx into psoriasis HPK, activates the expression of the transient receptor potential cation channel, subfamily C, member 6 (TRPC6), and promotes proper cell differentiation [34].

HL and HP display similar effects as the flavonoid luteolin-7-glucoside (LUT-7G) that increased the expression of the differentiation markers KRT1 and 10 in vitro in IL-22-stimulated HPK. Furthermore, LUT-7G inhibited IL-22-induced nuclear translocation of pSTAT3 in HPK [86]. CA, HL and HP were also able to inhibit the nuclear translocation of pSTAT3 and thus block the transcription of downstream genes that are involved in proliferation and cell cycle regulation [87]. The importance of STAT3 in the pathogenesis of psoriasis was demonstrated by the conditional STAT3 overexpression in mouse skin, leading to epidermal hyperplasia and the development of a psoriasis-like phenotype [88]. Although it was described that CA reduces the activation of the protein kinase AKT in glioblastoma cell lines [35], in our psoriasis setting, no reduction of AKT could be detected, although AKT is also involved in keratinocyte hyperproliferation and chronic inflammatory skin conditions [89]. The same was true for HL and HP (data not shown), so that these plant extracts obviously do not act on the AKT pathway.

To confirm the in vitro data, we generated a human ex vivo psoriasis model. Direct ex vivo use of psoriasis biopsies would be the best way to study the disease under controlled conditions. However, for this approach, several psoriasis biopsies from one patient would be required. As this raises practical and ethical problems, we used skin biopsies from reduction surgery and incubated them with the cytokine combination that we already used in the in vitro setting. It turned out that tape stripping of the skin represents an unspecific insult-like trauma that is essential for generating a proper psoriasis model. However, as KRT17 is also upregulated in wounds and by tape stripping, we could not use this marker in our ex vivo approach with skin biopsies.

The effect of HL, HP and CA on the release of pro-inflammatory cytokines like IL-6 and IL-8 was studied in our ex vivo psoriasis model. All three extracts reduced the expression of these pro-inflammatory cytokines. As CA (*Curcuma amada*) as well as turmeric (*Curcuma longa* L.) belong to the ginger family, their mode of action might be similar. Because the polyphenol curcumin isolated from turmeric is described as an anti-psoriatic agent that is used in TCM and in Ayurveda Medicine, a comparison to CA would be interesting. Curcumin downregulated pro-inflammatory cytokines such as IL-17 and TNF-α in IMQ-stimulated HaCaT cells [70]. However, the leading compound of CA is the diterpenoid LDD ((E)-Labda-8(17),12-diene-15,16-dial (LDD)) that is structurally different from the polyphenol curcumin. Nevertheless, CA and turmeric both show anti-inflammatory effects. Similar anti-inflammatory effects are displayed by luteolin and 3′,4′,5,7-tetramethoxyluteolin [86].

The most potent topical treatment for psoriasis is the anthracene derivative dithranol, formerly known as chrysarobin. It was originally obtained from the bark of the araroba tree that grows in the rain forest of the Amazon. Dithranol inhibits the release of pro-inflammatory cytokines and the proliferation of keratinocytes [46,81] and was used as a positive control in our settings. However, dithranol can cause severe skin irritations in vivo such as redness, itching and a burning sensation, and might stain the skin and clothes brown.

The anti-psoriatic effect of topical herbal therapies (e.g., *Aloe vera*, *Capsicum frutescens* or the alkaloid capsaicin, *Curcuma longa* or curcumin, *Hypericum perforatum*, Indigo naturalis, *Mahonia aquifolium*) has recently been evaluated in vivo [90,91,92,93,94,95,96,97]. Only a few extracts showed significant anti-psoriatic effects compared with control groups. One of the effective extracts originated from *Mahonia aquifolium* was used by native Americans for centuries to treat psoriasis. A randomized placebo-controlled double-blind study in 200 psoriasis patients confirmed the efficacy and safety of a 10% *Mahonia* ointment [95]. Another promising herbal treatment is Indigo naturalis (*Baphicacanthus cusia* Brem.)*,* an important medication in Traditional Chinese Medicine (TCM). It is a blue powder obtained from the plant *Baphicacanthus cusia* by grinding, fermentation and addition of lime. It contains 1.4% indigo and 0.16% indirubin. Treatment with indigo improved symptoms in a placebo-controlled study of 42 patients by 81%, while the improvement with placebo was only 26% [98]. As a side effect, 4 patients showed itching. However, Indigo naturalis may cause long-lasting blue staining of skin and clothes, and all studies with Indigo extract were performed with Asian patients. Whether the effect of Indigo naturalis is comparable in Caucasians has not yet been assessed [99].

Therefore, there is still a need for new topical treatment options for psoriasis. In our study, CA, native to Asia, displayed anti-inflammatory effects and reduced KRT17 expression in vitro, but in the ex vivo model, it had no effect on psoriasin and GLUT1 expression, and the reduction on BD2 expression was only small. In contrast, HL was effective in vitro and also strongly reduced the expression of psoriasin, BD2 and GLUT1 in the ex vivo skin model. This effect was even more prominent than for HP, that showed no effect on GLUT1 expression ex vivo. HL is easy to obtain in industrial countries because it is cultivated for beer production. Randomized placebo-controlled studies with HL in psoriasis are warranted to confirm the encouraging results of our in vitro and ex vivo studies. With HP, two small placebo-controlled, double-blind half-side comparisons with 10 or 20 patients have been performed. Patients with mild to moderate plaque psoriasis were treated with 5% HP extract and showed a significant reduction of the PASI and the lesional TNF-α expression [95,100].

## 5. Conclusions

We have established an in vitro and ex vivo psoriasis model by stimulating keratinocytes and tape-stripped skin biopsies with psoriasis-associated cytokines. In these models, we have identified three promising plant extracts—HL, HP and CA—with pronounced anti-psoriatic effects. The most promising extract is HL because of its anti-proliferative and anti-inflammatory effects at low concentrations in vitro and ex vivo. HL is available at relatively low prices on the market. It might be suitable for the topical long-term treatment of psoriasis. Used as a basic emollient, it might enhance the healing process and prolong the symptomless interval in psoriasis.

## Figures and Tables

**Table 1:** Characterization of 10 plant extracts used in folk medicines with their lead substances and dithranol as a control.

Plant Plant Family	Used in	Extract and Solubility	Lead Substance		Possible Anti-Psoriatic Effects
Asiatic pennywort (*Centella asiatica*) Apiaceae	TCM AM	HPE extract of dried leaves Soluble in PBS	Triterpenoid-Saponines: Asiaticoside (1.1%), Madecassosid (6.4%), DER 7/1	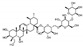 Asiaticoside	Anti-inflammatory [9,10] Anti-oxidant [10,11] Anti-proliferative [9,12] Wound healing induction [9,12]
Brahmi (*Bacopa monniera* L.) Scrophulariaceae	AM	HPE extract of dried leaves Soluble in ethanol (70%)	Steroid-Saponine: Bacosides (6.7%), DER 8/1	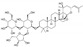 Bacoside A3	Anti-inflammatory [13] Anti-oxidant [13,14] Adaptogene [14]
Buckbean *(Menyanthes trifoliata* L.) Menyanthaceae	TCM	HPE extract of dried leaves Soluble in PBS	Secoiridoidglycoside: Dihydroforliamenthin (≈1%), DER: 2.5/1 (for 50% extract dissolved in ethanol)	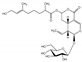	Anti-inflammatory [15] Anti-oxidant [15] Anti-proliferative [16] Inhibition of NO production [17]
Gentian (*Gentiana lutea*) Gentianaceae	OMM	HPE extract of roots Soluble in PBS	Bitter glycoside: Gentiopicroside (15.9%); DER ≈ 1.8/1 (for 50% extract dissolved in ethanol)	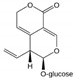	Anti-inflammatory [18,19,20,21] Anti-oxidative [18] Inhibition of NF-κB [18,20,21] Induction of ceramide synthesis [19]
Guggul (*Commiphora mukul*) Burceraceae	AM	Supercritical Co-solvent extract with CO_2_ + EtOH (95 + 5) of tree gum Soluble in ethanol (70%)	Phytosteroid: Guggulsterone (2%); DER: 3.5/1 (for 50% extract dissolved in MCT-oil)	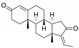	Anti-inflammatory [22,23,24] Inhibition of lipid peroxidation [23] Anti-proliferative [22,23,24] Inhibition of AKT + NF-κB [22,23,24] Inhibition of mTOR [25]
Hop (*Humulus lupulus*) Cannabaceae	OMM AM	Supercritical CO_2_ extract of dried hop cones Soluble in DMSO	Bitter hop acids: Humulones (48%), Lupulones (24%) DER ≈ 4/1	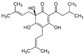	Anti-inflammatory [26,27,28] Anti-oxidant [26,27,28,29] Anti-proliferative [26,27,28] Inhibition of NF-κB + AP-1 [27]
St. John’s wort *(Hypericum* *perforatum*) Hypericaceae	OMM	Supercritical CO_2_ extract of dried branch tips with flowers Soluble in DMSO	Polyprenylated phloroglycinol derivative: Hyperforin (30%), DER ≈ 20/1	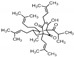	Anti-inflammatory [30,31] Anti-oxidant [30,31] Anti-proliferative [30,31] AMPK/mTOR inhibition [32] Differentiation [33,34]
Mango ginger (*Curcuma amada*) Zingiberaceae	AM	Supercritical CO_2_ extract of rhizoma Soluble in ethanol (100%)	Diterpenoid: (E)-Labda-8(17),12-diene-15,16-dial (LDD): 42%; DER: 33/1	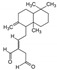	Anti-inflammatory [35] Anti-oxidative [36,37] Anti-proliferative [35,36,38] Inhibition of AKT [38] Pro-apoptotic [35,38]
Purple coneflower (*Echinacea purpurea*) Asteracea	NAM	Supercritical CO_2_ extract of the dried roots Soluble in ethanol (100%)	Total alkylamides (24.3%), total sterols: 4.8%, DER ≈ 125/1	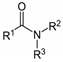	Anti-inflammatory [39,40] Anti-oxidative [40] Anti-proliferative [40] Inhibition of NF-κB [39,41] Promotion of DC maturation [41] Cannabinomimetics [39]
Sweet indraja (*Writhia tinctoria*) Apocynaceae	AM	Supercritical CO_2_ extract of seeds Soluble in DMSO	Triterpenes: α und β Amyrin: 0.7%, total sterols 5.4%, DER: 5.2/1	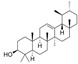 α-Amyrin	Anti-inflammatory [42,43,44,45] Anti-oxidative [42,45] Anti-ulcer activity [43,45] Anti-dandruff properties [45]
Dithranol originally from the Araroba tree *(Vataireopsis araroba*) Fabaceae		First synthesized in 1916 by the German pharmaceutical company Bayer Stock solution in DMSO	Anthracene compound: Dithranol (1,8-dihydroxy-9anthrone)	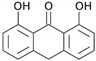	Anti-inflammatory [46] Antioxidant [46] Anti-proliferative [46] Useful in psoriasis [46] Side effects: e.g., skin irritation and discoloration of the skin

Abbreviations: AM: Ajurvedic Medicine, DC: Dendritic Cell, DER: Drug Extract Ratio, HPE: High-Pressure Ethanol extract, MTC: Medium-Chain Triglycerides, NAM: Native American Medicine, OMM: Occidental Monastic Medicine, TCM: Traditional Chinese Medicine.

**Table 2:** Anti-proliferative and anti-inflammatory potential of *Humulus lupulus*, *Hypericum perforatum* and *Curcuma amada*.

Plant Extract	Cell Viability HPK, EC_80_	Anti-Proliferative Effect Psoriasis-Like HPK, EC_50_	Inhibition of IL-6 Pso-Like HPK, EC_50_	Inhibition of IL-8 Pso-Like HPK, EC_50_
Hop (*Humulus lupulus*)	0.22 ± 1.15 µg/ml	0.36 ± 0.02 µg/ml	0.28 ± 0.26 µg/ml	0.34 ± 0.44 µg/ml
St John’s wort (*Hypericum perforatum*)	0.35 ± 1.08 µg/ml	0.70 ± 0.03 µg/ml	0.14 ± 0.12µg/ml	0.28 ± 0.32 µg/ml
Mango ginger (*Curcuma amada*)	2.12 ± 1.16 µg/ml	2.33 ± 0.63 µg/ml	1.08 ± 0.38 µg/ml	1.38 ± 0.67 µg/ml
**Control**				
Dithranol	0.05 ± 0.02 µg/ml	0.03 ± 0.001 µg/ml	0.01 ± 0.01 µg/ml	0.02 ± 0.01 µg/ml

Cells were treated with the corresponding extracts in a range from 0.2 to 400 µg/mL. For cell viability tests, HPK were incubated with the extracts for 24 h and the CellTiter-Glo2.0 Assay was performed (n = 3). For measurement of cell proliferation, psoriasis-like HPK were treated for 24 h with the corresponding extracts before the BrdU assay labeling was conducted (n = 6). The IL-6 and IL-8 protein expression level was measured by ELISA (n = 3) in the supernatant of psoriasis-like HPKs after 24 h extract treatment. EC_50_ (half maximal effective concentration) were calculated with the GraphPadPrism program.

## Data Availability

The data presented in this study are available on request from the corresponding author.

## Supplementary Materials

The following are available online at https://www.mdpi.com/2218-273X/11/3/371/s1, Table S1: Anti-proliferative and anti-inflammatory potential of 10 plant extracts used in folk medicines, Figure S1: Effect of psoriasis cytokines (cytokines (IL-17, IL-22. TNF-α, 20 ng/mL) in HPK on cell proliferation and cell cytotoxicity [100,101].

## References

[1] Melnikova I. (2009). Psoriasis market. Nat. Rev. Drug Discov..

[2] Benezeder T., Wolf P. (2019). Resolution of plaque-type psoriasis: What is left behind (and reinitiates the disease). Semin. Immunopathol..

[3] Hoffjan S., Stemmler S. (2007). On the role of the epidermal differentiation complex in ichthyosis vulgaris, atopic dermatitis and psoriasis. Br. J. Dermatol..

[4] Yang L., Fan X., Cui T., Dang E., Wang G. (2017). Nrf2 Promotes Keratinocyte Proliferation in Psoriasis through Up-Regulation of Keratin 6, Keratin 16, and Keratin 17. J. Investig. Dermatol..

[5] Herster F., Bittner Z., Archer N.K., Dickhöfer S., Eisel D., Eigenbrod T., Knorpp T., Schneiderhan-Marra N., Löffler M.W., Kalbacher H. (2020). Neutrophil extracellular trap-associated RNA and LL37 enable self-amplifying inflammation in psoriasis. Nat. Commun..

[6] Furue K., Yamamura K., Tsuji G., Mitoma C., Uchi H., Nakahara T., Kido-Nakahara M., Kadono T., Furue M. (2018). Highlighting Interleukin-36 Signalling in Plaque Psoriasis and Pustular Psoriasis. Acta Derm. Venereol..

[7] Lowes M.A., Russell C.B., Martin D.A., Towne J.E., Krueger J.G. (2013). The IL-23/T17 pathogenic axis in psoriasis is amplified by keratinocyte responses. Trends Immunol..

[8] Bai X., Yu C., Yang L., Luo Y., Zhi D., Wang G., Dang E. (2020). Anti-psoriatic properties of paeoniflorin: Suppression of the NF-kappaB pathway and Keratin 17. Eur. J. Dermatol. EJD.

[9] Sampson J., Raman A., Karlsen G., Navsaria H., Leigh I. (2001). In vitro keratinocyte antiproliferant effect of Centella asiatica extract and triterpenoid saponins. Phytomedicine.

[10] Liu M., Dai Y., Li Y., Luo Y., Huang F., Gong Z., Meng Q. (2008). Madecassoside Isolated fromCentella asiaticaHerbs Facilitates Burn Wound Healing in Mice. Planta Medica.

[11] Masola B., Oguntibeju O.O., Oyenihi A.B. (2018). Centella asiatica ameliorates diabetes-induced stress in rat tissues via influences on antioxidants and inflammatory cytokines. Biomed. Pharmacother..

[12] Bylka W., Znajdek-Awiżeń P., Studzińska-Sroka E., Dańczak-Pazdrowska A., Brzezińska M. (2014). Centella asiaticain Dermatology: An Overview. Phytother. Res..

[13] Vishnupriya P., Padma V.V. (2017). A Review on the Antioxidant and Therapeutic Potential of Bacopa monnieri. React. Oxyg. Species.

[14] Simpson T., Pase M.P., Stough C. (2015). Bacopa monnierias an Antioxidant Therapy to Reduce Oxidative Stress in the Aging Brain. Evidence-Based Complement. Altern. Med..

[15] Kowalczyk T., Sitarek P., Skała E., Toma M., Wielanek M., Pytel D., Wieczfińska J., Szemraj J., Śliwiński T. (2019). Induction of apoptosis by in vitro and in vivo plant extracts derived from *Menyanthes trifoliata* L. in human cancer cells. Cytotechnology.

[16] Kowalczyk T., Sitarek P., Skała E., Rijo P., Andrade J.M., Synowiec E., Szemraj J., Krajewska U., Śliwiński T. (2019). An Evaluation of the DNA-Protective Effects of Extracts from *Menyanthes trifoliata* L. Plants Derived from In Vitro Culture Associated with Redox Balance and Other Biological Activities. Oxidative Med. Cell. Longev..

[17] Zhu J.-J., Yang H.-X., Li Z.-H., Wang G.-K., Feng T., Liu J.-K. (2018). Anti-inflammatory lupane triterpenoids from Menyanthes trifoliata. J. Asian Nat. Prod. Res..

[18] Xie X., Li H., Wang Y., Wan Z., Luo S., Zhao Z., Liu J., Wu X., Li X., Li X. (2019). Therapeutic effects of gentiopicroside on adjuvant-induced arthritis by inhibiting inflammation and oxidative stress in rats. Int. Immunopharmacol..

[19] Gendrisch F., Nováčková A., Sochorová M., Haarhaus B., Vávrová K., Schempp C.M., Wölfle U. (2020). Gentiana lutea Extract Modulates Ceramide Synthesis in Primary and Psoriasis-Like Keratinocytes. Molecules.

[20] Xiao H., Sun X., Liu R., Chen Z., Lin Z., Yang Y., Zhang M., Liu P., Quan S., Huang H. (2020). Gentiopicroside activates the bile acid receptor Gpbar1 (TGR5) to repress NF-kappaB pathway and ameliorate diabetic nephropathy. Pharmacol. Res..

[21] Wang Q., Zhou X., Yang L., Luo M., Han L., Lu Y., Shi Q., Wang Y., Liang Q. (2019). Gentiopicroside (GENT) protects against sepsis induced by lipopolysaccharide (LPS) through the NF-κB signaling pathway. Ann. Transl. Med..

[22] Bhat A.A., Prabhu K.S., Kuttikrishnan S., Krishnankutty R., Babu J., Mohammad R.M., Uddin S. (2017). Potential therapeutic targets of Guggulsterone in cancer. Nutr. Metab..

[23] Shishodia S., Azu N., Rosenzweig J.A., Jackson D.A. (2015). Guggulsterone for Chemoprevention of Cancer. Curr. Pharm. Des..

[24] Kunnumakkara A.B., Banik K., Bordoloi D., Harsha C., Sailo B.L., Padmavathi G., Roy N.K., Gupta S.C., Aggarwal B.B. (2018). Googling the Guggul (Commiphora and Boswellia) for Prevention of Chronic Diseases. Front. Pharmacol..

[25] Ramachandran C., Nair S.M., Quirrin K.-W., Melnick S.J. (2013). Hypolipidemic Effects of a Proprietary Commiphora Mukul Gum Resin Extract and Medium-Chain Triglyceride Preparation (GU-MCT810). J. Evid. Based Integr. Med..

[26] Chen W., Becker T., Qian F., Ring J. (2013). Beer and beer compounds: Physiological effects on skin health. J. Eur. Acad. Dermatol. Venereol..

[27] Van Cleemput M., Cattoor K., de Bosscher K., Haegeman G., de Keukeleire D., Heyerick A. (2009). Hop (*Humulus lupulus*)-Derived Bitter Acids as Multipotent Bioactive Compounds. J. Nat. Prod..

[28] Kawanishi S., Oikawa S., Murata M. (2005). Evaluation for Safety of Antioxidant Chemopreventive Agents. Antioxid. Redox Signal..

[29] Weber N., Biehler K., Schwabe K., Haarhaus B., Quirin K.-W., Frank U., Schempp C.M., Wölfle U. (2019). Hop Extract Acts as an Antioxidant with Antimicrobial Effects against *Propionibacterium acnes* and *Staphylococcus aureus*. Molecules.

[30] Wölfle U., Seelinger G., Schempp C. (2013). Topical Application of St. John’s Wort (*Hypericum perforatum*). Planta Med..

[31] Sevastre-Berghian A.C., Toma V.A., Sevastre B., Hanganu D., Vlase L., Benedec D., Oniga I., Baldea I., Olteanu D., Moldovan R. (2019). Characterization and biological effects of Hypericum extracts on experimentally-induced—Anxiety, oxidative stress and inflammation in rats. J. Physiol. Pharmacol. Off. J. Pol. Physiol. Soc..

[32] You M.-K., Kim H.-J., Kook J.H., Kim H.-A. (2018). St. John’s Wort Regulates Proliferation and Apoptosis in MCF-7 Human Breast Cancer Cells by Inhibiting AMPK/mTOR and Activating the Mitochondrial Pathway. Int. J. Mol. Sci..

[33] Müller M., Essin K., Hill K., Beschmann H., Rubant S.A., Schempp C.M., Gollasch M., Boehncke W.-H., Harteneck C., Müller W.E. (2008). Specific TRPC6 Channel Activation, a Novel Approach to Stimulate Keratinocyte Differentiation. J. Biol. Chem..

[34] Leuner K., Kraus M., Woelfle U., Beschmann H., Harteneck C., Boehncke W.-H., Schempp C.M., Müller W.E. (2011). Reduced TRPC Channel Expression in Psoriatic Keratinocytes Is Associated with Impaired Differentiation and Enhanced Proliferation. PLoS ONE.

[35] Ramachandran C., Portalatin G., Quirin K.-W., Escalon E., Khatib Z., Melnick S.J. (2015). Inhibition of AKT signaling by supercritical CO2 extract of mango ginger (*Curcuma amada Roxb*.) in human glioblastoma cells. J. Complement. Integr. Med..

[36] Win N.N., Ito T., Ngwe H., Win Y.Y., Prema, Okamoto Y., Tanaka M., Asakawa Y., Abe I., Morita H. (2017). Labdane diterpenoids from Curcuma amada rhizomes collected in Myanmar and their antiproliferative activities. Fitoterapia.

[37] Jatoi S.A., Kikuchi A., Gilani S.A., Watanabe K.N. (2007). Phytochemical, pharmacological and ethnobotanical studies in mango ginger (*Curcuma amada Roxb*.; Zingiberaceae). Phytotherapy Res..

[38] Ramachandran C., Quirin K.-W., Escalon E.A., Lollett I.V., Melnick S.J. (2015). Therapeutic Effect of Supercritical CO2 Extracts of Curcuma Species with Cancer Drugs in Rhabdomyosarcoma Cell Lines. Phytotherapy Res..

[39] Raduner S., Majewska A., Chen J.-Z., Xie X.-Q., Hamon J., Faller B., Altmann K.-H., Gertsch J. (2006). Alkylamides from Echinacea Are a New Class of Cannabinomimetics. J. Biol. Chem..

[40] Aarland R.C., Bañuelos-Hernández A.E., Fragoso-Serrano M., Sierra-Palacios E.D.C., de León-Sánchez F.D., Pérez-Flores L.J., Rivera-Cabrera F., Mendoza-Espinoza J.A. (2017). Studies on phytochemical, antioxidant, anti-inflammatory, hypoglycaemic and antiproliferative activities of Echinacea purpurea and Echinacea angustifolia extracts. Pharm. Biol..

[41] Li Y., Wang Y., Wu Y., Wang B., Chen X., Xu X., Chen H., Li W., Xu X. (2017). Echinacea pupurea extracts promote murine dendritic cell maturation by activation of JNK, p38 MAPK and NF-κB pathways. Dev. Comp. Immunol..

[42] Nath S., Pathak B. Phytochemical and Pharmacological Characteristics of Wrightiatinctoria: A Review. /paper/Phytochemical-and-Pharmacological-Characteristics-%3A-Nath-Pathak/9f0ce9270365efb25ed698eeeff06c4e920ee67a.

[43] Srivastava R. (2014). A review on phytochemical, pharmacological, and pharmacognostical profile of Wrightia tinctoria: Adulterant of kurchi. Pharmacogn. Rev..

[44] Babu M. (2015). Pharmacological Evaluation of Wrightia Tinctoria—A Review. Sci. Rep..

[45] Rao B., Rajeswari D., Devarakonda R., Battu H. (2019). Phytochemical and Pharmacological Studies on Wrightia Tinctoria. World J. Pharm. Pharm. Sci..

[46] Sehgal V.N., Verma P., Khurana A. (2014). Anthralin/dithranol in dermatology. Int. J. Dermatol..

[47] Ghosh S., Hayden M.S. (2008). New regulators of NF-κB in inflammation. Nat. Rev. Immunol..

[48] Singh S., Singh R., Banerjee S., Negi A.S., Shanker K. (2012). Determination of anti-tubercular agent in mango ginger (*Curcuma amada Roxb*.) by reverse phase HPLC-PDA-MS. Food Chem..

[49] Rheinwatd J.G., Green H. (1975). Seria cultivation of strains of human epidemal keratinocytes: The formation keratinizin colonies from single cell is. Cell.

[50] Schmittgen T.D., Livak K.J. (2008). Analyzing real-time PCR data by the comparative CT method. Nat. Protoc..

[51] Pfaff C.M., Marquardt Y., Fietkau K., Baron J.M., Lüscher B. (2017). The psoriasis-associated IL-17A induces and cooperates with IL-36 cytokines to control keratinocyte differentiation and function. Sci. Rep..

[52] Muromoto R., Hirao T., Tawa K., Hirashima K., Kon S., Kitai Y., Matsuda T. (2016). IL-17A plays a central role in the expression of psoriasis signature genes through the induction of IκB-ζ in keratinocytes. Int. Immunol..

[53] Teunissen M.B., Bos J.D., Koomen C.W., Malefyt R.D.W., Wierenga E.A. (1998). Interleukin-17 and Interferon-γ Synergize in the Enhancement of Proinflammatory Cytokine Production by Human Keratinocytes. J. Investig. Dermatol..

[54] Wolk K., Haugen H.S., Xu W., Witte E., Waggie K., Anderson M., Baur E.V., Witte K., Warszawska K., Philipp S. (2009). IL-22 and IL-20 are key mediators of the epidermal alterations in psoriasis while IL-17 and IFN-γ are not. J. Mol. Med..

[55] Ettehadi P., Greaves M.W., Wallach D., Aderka D., Camp R.D.R. (2008). Elevated tumour necrosis factor-alpha (TNF-α) biological activity in psoriatic skin lesions. Clin. Exp. Immunol..

[56] De Jongh G.J., Zeeuwen P.L., Kucharekova M., Pfundt R., van der Valk P.G., Blokx W., Dogan A., Hiemstra P.S., van de Kerkhof P.C., Schalkwijk J. (2005). High Expression Levels of Keratinocyte Antimicrobial Proteins in Psoriasis Compared with Atopic Dermatitis. J. Investig. Dermatol..

[57] Kolbinger F., Loesche C., Valentin M.-A., Jiang X., Cheng Y., Jarvis P., Peters T., Calonder C., Bruin G., Polus F. (2017). β-Defensin 2 is a responsive biomarker of IL-17A–driven skin pathology in patients with psoriasis. J. Allergy Clin. Immunol..

[58] Zanet J., Freije A., Ruiz M., Coulon V., Sanz J.R., Chiesa J., Gandarillas A. (2010). A Mitosis Block Links Active Cell Cycle with Human Epidermal Differentiation and Results in Endoreplication. PLoS ONE.

[59] Zhang Z., Zi Z., Lee E.E., Zhao J., Contreras D.C., South A.P., Abel E.D., Chong B.F., Vandergriff T., Hosler G.A. (2018). Differential glucose requirement in skin homeostasis and injury identifies a therapeutic target for psoriasis. Nat. Med..

[60] Hodeib A.A.-H., Neinaa Y.M.-H., Zakaria S.S., Alshenawy H.A.-S. (2018). Glucose transporter-1 (GLUT-1) expression in psoriasis: Correlation with disease severity. Int. J. Dermatol..

[61] Hiebert P., Werner S. (2018). Targeting metabolism to treat psoriasis. Nat. Med..

[62] Neuner P., Urbanski A., Trautinger F., Möller A., Kirnbauer R., Kapp A., Schöpf E., Schwarz T., Luger T.A. (1991). Increased IL-6 Production by Monocytes and Keratinocytes in Patients with Psoriasis. J. Investig. Dermatol..

[63] Gillitzer R., Berger R., Mielke V., Müller C., Wolff K., Stingl G. (1991). Upper Keratinocytes of Psoriatic Skin Lesions Express High Levels of NAP-1/IL-8 mRNA In Situ. J. Investig. Dermatol..

[64] Mathes S.H., Ruffner H., Graf-Hausner U. (2014). The use of skin models in drug development. Adv. Drug Deliv. Rev..

[65] D’Amico F., Trovato C., Skarmoutsou E., Rossi G.A., Granata M., Longo V., Gangemi P., Pettinato M., Mazzarino M.C. (2015). Effects of adalimumab, etanercept and ustekinumab on the expression of psoriasin (S100A7) in psoriatic skin. J. Dermatol. Sci..

[66] Zhang X., Yin M., Zhang L.-J. (2019). Keratin 6, 16 and 17—Critical Barrier Alarmin Molecules in Skin Wounds and Psoriasis. Cells.

[67] Lee S.Y., Nam S., Hong I.K., Kim H., Yang H., Cho H.-J. (2018). Antiproliferation of keratinocytes and alleviation of psoriasis by the ethanol extract of Artemisia capillaris. Phytother. Res..

[68] Thaçi D., Augustin M., Krutmann J., Luger T. (2015). Importance of basic therapy in psoriasis. J. Dtsch. Dermatol. Ges..

[69] Varma S.R., Sivaprakasam T.O., Mishra A., Prabhu S. (2017). Imiquimod-induced psoriasis-like inflammation in differentiated Human keratinocytes: Its evaluation using curcumin. Eur. J. Pharmacol..

[70] Sun J., Zhao Y., Hu J. (2013). Curcumin Inhibits Imiquimod-Induced Psoriasis-Like Inflammation by Inhibiting IL-1beta and IL-6 Production in Mice. PLoS ONE.

[71] Lin Y.-K., Leu Y.-L., Yang S.-H., Chen H.-W., Wang C.-T., Pang J.-H.S. (2009). Anti-psoriatic effects of indigo naturalis on the proliferation and differentiation of keratinocytes with indirubin as the active component. J. Dermatol. Sci..

[72] Jeon Y.-J., Sah S.K., Yang H.S., Lee J.H., Shin J., Kim T.-Y. (2017). Rhododendrin inhibits toll-like receptor-7-mediated psoriasis-like skin inflammation in mice. Exp. Mol. Med..

[73] Li H.-J., Wu N.-L., Lee G.-A., Hung C.-F. (2018). The Therapeutic Potential and Molecular Mechanism of Isoflavone Extract against Psoriasis. Sci. Rep..

[74] Soboleva A.G., Zolotarenko A.D., Sobolev V.V., Bruskin S.A., Piruzian E.S., Mezentsev A.V. (2014). Genetically predetermined limitation in HaCaT cells that affects their ability to serve as an experimental model of psoriasis. Russ. J. Genet..

[75] Leigh I., Navsaria H., Purkis P., McKay I., Bowden P., Riddle P. (1995). Keratins (Kl6 and Kl7) as markers of keratinocyte hyperproliferation in psoriasis in vivo and in vitro. Br. J. Dermatol..

[76] Zhang W., Dang E., Shi X., Jin L., Feng Z., Hu L., Wu Y., Wang G. (2012). The Pro-Inflammatory Cytokine IL-22 Up-Regulates Keratin 17 Expression in Keratinocytes via STAT3 and ERK1/2. PLoS ONE.

[77] Shi X., Jin L., Dang E., Chang T., Feng Z., Liu Y., Wang G. (2011). IL-17A Upregulates Keratin 17 Expression in Keratinocytes through STAT1- and STAT3-Dependent Mechanisms. J. Investig. Dermatol..

[78] De Jong E.M.G.J., van Vlijmen I.M.M.J., van Erp P.E.J., Ramaekers F.C.S., Troyanovski S.M., van de Kerkhof P.C.M. (1991). Keratin 17: A useful marker in anti-psoriatic therapies. Arch. Dermatol. Res..

[79] Radoja N., Komine M., Jho S.H., Blumenberg M., Tomic-Canic M. (2000). Novel Mechanism of Steroid Action in Skin through Glucocorticoid Receptor Monomers. Mol. Cell. Biol..

[80] Gilfix B.M., Eckert R.L. (1985). Coordinate control by vitamin A of keratin gene expression in human keratinocytes. J. Biol. Chem..

[81] Bonnekoh B., Böckelmann R., Ambach A., Gollnick H. (2001). Dithranol and Dimethylfumarate Suppress the Interferon-γ-Induced Up-Regulation of Cytokeratin 17 as a Putative Psoriasis Autoantigen in vitro. Ski. Pharmacol. Physiol..

[82] Jin L., Wang G. (2014). Keratin 17: A Critical Player in the Pathogenesis of Psoriasis. Med. Res. Rev..

[83] Chang T., Sun L., Wang Y., Wang D., Li W., Li C., Gao T., Liu Y., Wang G. (2011). Inhibition of keratin 17 expression with antisense and RNAi strategies: Exploring novel therapy for psoriasis. Exp. Dermatol..

[84] Zhang J., Li X., Wei J., Chen H., Lu Y., Li L., Han L., Lu C. (2018). Gallic acid inhibits the expression of keratin 16 and keratin 17 through Nrf2 in psoriasis-like skin disease. Int. Immunopharmacol..

[85] Niyonsaba O., Ushio H., Nakano N., Ng W., Sayama K., Hashimoto K., Nagaoka I. (2007). Antimicrobial Peptides Human β-Defensins Stimulate Epidermal Keratinocyte Migration, Proliferation and Production of Proinflammatory Cytokines and Chemokines. J. Investig. Dermatol..

[86] Palombo R., Savini I., Avigliano L., Madonna S., Cavani A., Albanesi C., Mauriello A., Melino G., Terrinoni A. (2016). Luteolin-7-glucoside inhibits IL-22/STAT3 pathway, reducing proliferation, acanthosis, and inflammation in keratinocytes and in mouse psoriatic model. Cell Death Dis..

[87] Zheng Y., Danilenko D.M., Valdez P.A., Kasman I., Eastham-Anderson J., Wu J., Ouyang W. (2006). Interleukin-22, a TH17 cytokine, mediates IL-23-induced dermal inflammation and acanthosis. Nat. Cell Biol..

[88] Sano S., Chan K.S., Carbajal S., Clifford J.L., Peavey M., Kiguchi K., Itami S., Nickoloff B.J., di Giovanni J. (2005). Stat3 links activated keratinocytes and immunocytes required for development of psoriasis in a novel transgenic mouse model. Nat. Med..

[89] Zhang M., Zhang X. (2019). The role of PI3K/AKT/FOXO signaling in psoriasis. Arch. Dermatol. Res..

[90] Gupta R., Gupta M., Mangal S., Agrawal U., Vyas S.P. (2014). Capsaicin-loaded vesicular systems designed for enhancing localized delivery for psoriasis therapy. Artif. Cell Nanomed. Biotechnol..

[91] Choonhakarn C., Busaracome P., Sripanidkulchai B., Sarakarn P. (2010). A prospective, randomized clinical trial comparing topical aloe vera with 0.1% triamcinolone acetonide in mild to moderate plaque psoriasis. J. Eur. Acad. Dermatol. Venereol..

[92] Di Nardo V., Gianfaldoni S., Tchernev G., Wollina U., Barygina V., Lotti J., Daaboul F., Lotti T. (2018). Use of Curcumin in Psoriasis. Open Access Maced. J. Med. Sci..

[93] Hashemian F., Mansouri P., Mirafzal S., Najafizadeh P., Safaei-Naraghi Z., Salehi-Surmaghi M. (2017). The impact of topical Saint John’s Wort (*Hypericum perforatum*) treatment on tissue tumor necrosis factor-alpha levels in plaque-type psoriasis: A pilot study. J. Postgrad. Med..

[94] Lin Y.-K., See L.-C., Huang Y.-H., Chang Y.-C., Tsou T.-C., Lin T.-Y., Lin N.-L. (2014). Efficacy and safety of Indigo naturalis extract in oil (Lindioil) in treating nail psoriasis: A randomized, observer-blind, vehicle-controlled trial. Phytomedicine.

[95] Bernstein S., Donsky H., Gulliver W., Hamilton D., Nobel S., Norman R. (2006). Treatment of Mild to Moderate Psoriasis with Reliéva, a Mahonia aquifolium Extract—A Double-Blind, Placebo-Controlled Study. Am. J. Ther..

[96] Herman A., Herman A.P. (2016). Topically Used Herbal Products for the Treatment of Psoriasis—Mechanism of Action, Drug Delivery, Clinical Studies. Planta Med..

[97] Hoffmann J., Gendrisch F., Schempp C.M., Wölfle U. (2020). New Herbal Biomedicines for the Topical Treatment of Dermatological Disorders. Biomedicines.

[98] Lin Y.-K., Chang C.-J., Chang Y.-C., Wong W.-R., Chang S.-C., Pang J.-H.S. (2008). Clinical Assessment of Patients with Recalcitrant Psoriasis in a Randomized, Observer-Blind, Vehicle-Controlled Trial Using Indigo Naturalis. Arch. Dermatol..

[99] Najafizadeh P., Hashemian F., Mansouri P., Farshi S., Surmaghi M.S., Chalangari R. (2012). The evaluation of the clinical effect of topical St Johns wort (*Hypericum perforatum* L.) in plaque type psoriasis vulgaris: A pilot study. Australas. J. Dermatol..

[100] Hawley-Nelson P., Vousden K.H., Hubbert N.L., Lowy D.R., Schiller J.T. (1989). HPV16 E6 and E7 Proteins Cooperate to Immortalize Human Foreskin Keratinocytes. EMBO J..

[101] Sprenger A., Küttner V., Biniossek M.L., Gretzmeier C., Boerries M., Mack C., Has C., Bruckner-Tuderman L., Dengjel J. (2010). Comparative Quantitation of Proteome Alterations Induced by Aging or Immortalization in Primary Human Fibroblasts and Keratinocytes for Clinical Applications. Mol. BioSyst..

