# Supplementary material for: The Effect of Herbal Medicinal Products on Psoriasis-Like Keratinocytes
Source: Biomolecules. 2021 Mar 2;11(3):371. doi: 10.3390/biom11030371 (PMC8000521; doi:10.3390/biom11030371)
Supplement: Supplementary file 1 [file biomolecules-11-00371-s001.pdf]

Supplementary Material

Table S1: Anti-proliferative and anti-inflammatory potential of 10 plant extracts used in folk medicines.

Plant extract	Cell viability HPK, EC_{80}	Anti-proliferative effect Pso-like NHK.1, EC_{50}	Inhibition of IL-6 Pso-like HPK, EC_{50}	Inhibition of IL-8 Pso-like HPK, EC_{50}
Asiatic pennywort (*Centella asiatica*)	168.5 ± 4.33 µg/ml	198.80 ± 1.71 µg/ml	no anti-inflammatory effect	133.90 ± 1.51 µg/ml
Brahmi (*Bacopa monniera* L.)	84.02 ± 1.35 µg/ml	158.80 ± 1.15 µg/ml	38.21 ± 1.12 µg/ml	66.36 ± 1.16 µg/ml
Buckbean (*Menyanthes trifoliata* L.)	73.7 ± 4.44 µg/ml	34.75 ± 1.14 µg/ml	5.16 ± 1.75 µg/ml	6.43 ± 2.91 µg/ml
Gentian (*Gentiana lutea*)	187.8 ± 1.35 µg/ml	229.00 ± 1.41 µg/ml	no anti-inflammatory effect	303.60 ± 1.39 µg/ml
Guggul (*Commiphora mukul*)	25.83 ± 1.22 µg/ml	24.97 ± 1.60 µg/ml	11.53 ± 2.32 µg/ml	14.62 ± 1.43 µg/ml
Hop (*Humulus lupulus*)	0.22 ± 1.15 µg/ml	0.20 ± 1.14 µg/ml	0.38 ± 0.01 µg/ml	0.84 ± 0.14 µg/ml
St John's wort (*Hypericum perforatum)*	0.35 ± 1.08 µg/ml	0.69 ± 1.06 µg/ml	0.34 ± 0.23 µg/ml	0.65 ± 0.004 µg/ml
Mango ginger (*Curcuma amada)*	2.12 ± 1.16 µg/ml	3.95 ± 1.06 µg/ml	2.12 ± 1.09 µg/ml	2.08 ± 1.08 µg/ml
Purple coneflower (*Echinacea purpura*)	61.36 ± 1.18 µg/ml	227.10 ± 1.98 µg/ml	55.67 ± 6.32 µg/ml	4.85 ± 1.38 µg/ml
Sweet indraja (*Wrightia tinctoria*)	no effect on cell viability until 400 µg/ml	no anti-proliferative effect	133.30 ± 1.08 µg/ml	124.30 µg/ml ± 1.28 µg/ml
**Controls**				
Dithranol	0.03 ± 0.01 µg/ml	0.07 ± 0.19	0.02 ± 0.001 µg/ml	0.01 ± 0.01 µg/ml

Cells were treated with the corresponding extracts in a range from 0.2 to 400 µg/mL. For cell viability tests, HPKs were incubated with the extracts for 24 h and the CellTiter-Glo2.0 Assay was performed (n = 3). For measurement of cell proliferation, psoriasis-like immortalized HPK (iHPK) were generated from iHPK [1,2] using IL-17A, IL-22 and TNF-α. These cells were then treated for 24 h with the corresponding extracts (n = 3). The IL-6 and IL-8 protein level was measured by ELISA in the supernatant of psoriasis-like HPKs after 24 h extract treatment (n = 3, technical replicates). EC_{50} (half-maximal effective concentration) and EC_{80} (in the cell viability assay showing 80% viable cells) were calculated using GraphPad Prism.

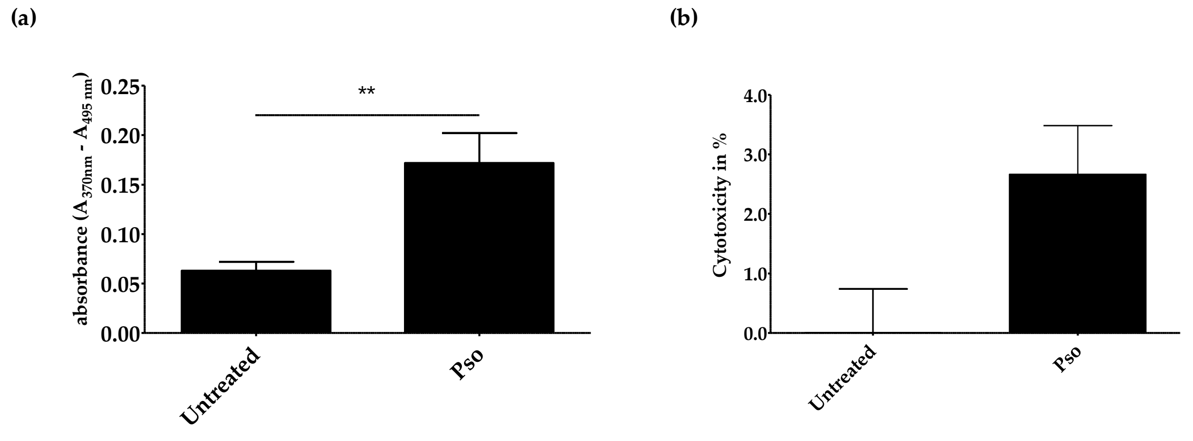

Figure S1. Effect of psoriasis cytokines (cytokines (IL-17, IL-22. TNF-α, 20 ng/mL) in HPK on cell proliferation and cell cytotoxicity. (**a**) Cell proliferation was measured in untreated HPK or psoriasis-like HPK (HPK treated with psoriasis cytokines for 72 h; Pso) with the BrdU assay (n = 6). (**b**) Cytotoxicity was measured in the cell supernatant of untreated HPK and psoriasis-like HPK (Pso) with the LDH kit from Roche according to the manufacturer's instructions (n = 3).

The treatment with psoriasis cytokines shows a statistically significant increase in cell proliferation with less than 4% cell toxicity, so that the cytotoxicity can be neglected.

References

1. Sprenger, A.; Küttner, V.; Biniossek, M.L.; Gretzmeier, C.; Boerries, M.; Mack, C.; Has, C.; Bruckner-Tuderman, L.; Dengjel, J. Comparative Quantitation of Proteome Alterations Induced by Aging or Immortalization in Primary Human Fibroblasts and Keratinocytes for Clinical Applications. *Mol. BioSyst.* **2010**, *6*, 1579–1582, doi:10.1039/C003962D.
2. Hawley-Nelson, P.; Vousden, K.H.; Hubbert, N.L.; Lowy, D.R.; Schiller, J.T. HPV16 E6 and E7 Proteins Cooperate to Immortalize Human Foreskin Keratinocytes. *EMBO J.* **1989**, *8*, 3905–3910.
